# Hashimoto's Thyroiditis Encephalopathy Induced by COVID-19 Infection

**DOI:** 10.7759/cureus.28419

**Published:** 2022-08-26

**Authors:** Misbahuddin Khaja, Zaheer A Qureshi, Kazi Samsuddoha, Vikram Itare, Petr Stastka, Jaydeep Mahasamudram, Faryal Altaf, Arundhati Dileep

**Affiliations:** 1 Internal Medicine/Pulmonary Critical Care, Icahn School of Medicine at Mount Sinai/BronxCare Health System, New York City, USA; 2 Internal Medicine, St. Vincent Medical Center, Bridgeport, USA; 3 Internal Medicine, BronxCare Health System, New York City, USA; 4 Internal Medicine, American University of the Caribbean School of Medicine, Cupecoy, SXM; 5 Pulmonary Medicine, BronxCare Health System, New York City, USA

**Keywords:** autoimmune, thyroid, viral disease, hashimoto’s encephalopathy, covid-19 outbreak, covid 19, coronavirus, thyroiditis, hashimoto

## Abstract

Various factors can lead to thyroiditis, including any acute inflammatory process, especially viral illness. While coronavirus disease 2019 (COVID-19) has been linked to disorders of various systems, there is a lack of literature showing an association of coronavirus with the cause of Hashimoto's thyroiditis. Several possible mechanisms for this outcome have been proposed; chief among them is molecular mimicry. Here, we are reporting a case of Hashimoto's thyroiditis incited by COVID-19 in a 34-year-old obese female who presented with anxiety, behavioral changes, and repeated head movements. The patient had an elevated thyroid stimulating hormone (TSH) level, a low thyroxine (T4) level, and a positive anti-microsomal antibody screen. The patient also tested positive for severe acute respiratory syndrome coronavirus 2 (SARS-CoV-2) RNA. Ultrasound of the patient's neck showed an enlarged heterogeneous thyroid gland. Thyroid replacement therapy with intravenous levothyroxine was started with the subsequent oral transition. Concurrently, she received antibiotics, steroids, and low-molecular-weight heparin for COVID-19. The patient exhibited significant improvement in her mental status, with an eventual return to baseline. The results of the thyroid panel obtained at the outpatient follow-up were normal. Although there is a paucity of data to show COVID-19 as a cause of this painless thyroiditis, this case demonstrates such causality between these two.

## Introduction

Multiple factors predispose one to thyroiditis including viral and bacterial infections, radiation, certain drugs (e.g. amiodarone-induced thyroiditis), and vigorous palpation. These also include genetic susceptibility and environmental stressors. Acute inflammatory processes can result in thyroid disorders, especially viral illnesses [1]. The mechanism for this link is unclear, but the existing literature on coronavirus disease 2019 (COVID-19)-mediated autoimmune processes suggests several likely possibilities: bystander activation, molecular mimicry, presentation of cryptic antigens, cross-reaction, or epitope spreading [1].

Although COVID-19 can cause this disease process, it is essential to note that most individuals don't manifest signs of a thyroid disorder. However, those who do manifest signs report anterior neck tenderness/pain, fatigue, weight changes, constipation, diarrhea, or other classic thyroiditis-associated signs or symptoms. The literature has established the possibility of different viral illnesses as causes of thyroiditis, but there is a lack of evidence showing coronavirus as a possible cause of Hashimoto's thyroiditis. COVID-19 can induce autoimmunity and lead to autoimmune hemolytic anemia (AIHA), Guillain‐Barré syndrome (GBS), immune thrombocytopenic purpura, and Kawasaki disease, or autoimmune thyroid diseases including Hashimoto's [1]. Few articles have shown COVID-19 infection to be causing subacute thyroiditis and short-term reversible thyroid dysfunction [2-5].

## Case presentation

**Figure 1:**
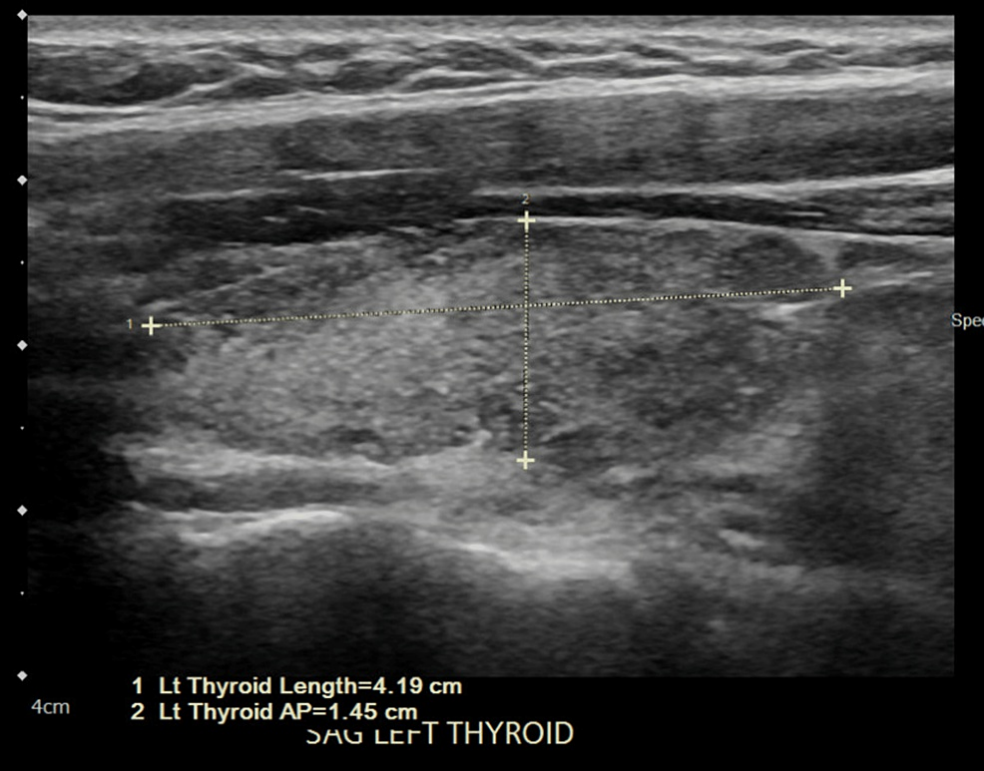
Ultrasound of the right thyroid lobe showing an enlarged heterogeneous thyroid gland

**Figure 2:**
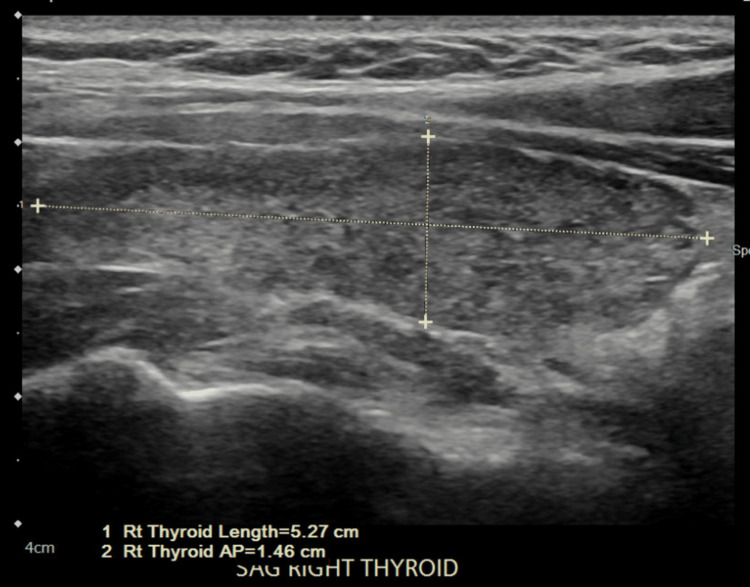
Ultrasound of the right thyroid lobe showing an enlarged heterogeneous thyroid gland

A 34-year-old obese female with a body mass index (BMI) of 36 presented to the emergency department with acute behavioral changes. In the emergency department, she was noted to have signs of generalized anxiety with accompanying repetitive head movements. She had a history of asthma (never intubated, not on steroids), morbid obesity, and galactorrhea. Her family history was significant for hypertension and diabetes mellitus (DM) in multiple family members. She denied chest pain, palpitations, nausea, vomiting, fever, malaise, and recent flu-like symptoms. Physical examination was normal, except for the aforementioned repetitive head movement. On admission, her thyroid stimulating hormone (TSH) level was 47.10 mIU/L, T3 was 34 ng/dL, and T4 was 0.15 μg/dL. Her anti-microsomal antibody test came out positive. Her lab values showed the D-dimer level at <150 ng/mL, lactate dehydrogenase (LDH) at 326 units/L, ferritin level at 46 ng/mL, creatine kinase at 1154 mcg/L, and lactic acid level at 3.6 mmol/L (Table 1). She tested positive for severe acute respiratory syndrome coronavirus 2 (SARS-CoV-2), although she did not have any signs or symptoms of COVID-19. The chest X-ray did not reveal any acute pathology, and computed tomography (CT) of the head was unremarkable. Ultrasound of the neck showed an enlarged heterogeneous thyroid gland, which limited the evaluation of the underlying parenchyma. The right and left thyroid lobes are shown in Figures 1, 2, respectively. There was no measurable demonstrated solid, cystic, or complex nodule. MRI of the head did not show any pituitary adenoma. She had no prior history of thyroid ultrasound or thyroid function tests.

**Table 1:** Initial laboratory results

	Value	Normal range
Thyroid stimulating hormone (TSH)	58.60	0.40-4.50 mlU/L
Triiodothyronine (T3)	34	60-181 ng/dL
Thyroxine (T4)	0.15	4.8-10.4 μg/dL
Microsomal antibody	296	≤35.0 IU/mL
Thyroid peroxidase	>900	<9 IU/mL
Coronavirus RNA	Detected	Detected/non-detected
D-dimer	<150	0-230 ng/mL
Lactate dehydrogenase	326	100-190 units/L
Ferritin	46	13-150 ng/mL
Creatine kinase	1154	20-200 units/L

The patient was initially started on intravenous levothyroxine and subsequently transitioned to an oral 200 mcg dose. She also received ceftriaxone, dexamethasone, and prophylactic anticoagulation for COVID-19. Due to encephalopathy developed during the hospital course, she required multiple doses of haloperidol. After thyroid replacement was initiated, her mental status improved drastically and ultimately returned to baseline. Her thyroid panel was later repeated at the outpatient follow-up and showed normal values (Table 2).

**Table 2:** Thyroid panel trend Free T4, free thyroxine (normal 0.80-2.0 ng/dL); T3, triiodothyronine (normal 60-181 ng/dL); TSH, thyroid stimulating hormone (normal 0.40-4.5 mIU/L)

	02/11/2019	05/07/2020	05/08/2020	05/12/2020	06/23/2020	12/02/2020
Free T4 (μg/dL)	1.06	0.15	0.30	0.63	1.0	1.03
T3 (ng/dL)	124	34	34	68	101	128
TSH (mIU/L)	2.74	47.10	58.60	51.30	3.58	4.11

## Discussion

Thyroiditis can be classified as silent or subclinical thyroiditis, transient hyperthyroidism, acute or subacute infectious thyroiditis, and chronic autoimmune thyroiditis including Graves' disease and Hashimoto's thyroiditis [6]. Antithyroid antibody tests that include thyroid peroxidase autoantibodies (TPOs), TSH receptor autoantibodies, and thyroglobulin autoantibodies may aid in confirming these diseases [7]. These autoimmune markers are not necessarily specific, but if they are positive and the other possible diagnoses are being excluded, then the suggested identifiable diagnosis by the specificity of the test is favored. Clinical manifestations, in addition to laboratory and histopathology findings, vary between the different thyroid disorders. Specifically, Hashimoto's thyroiditis is well known to be painless and for this very reason, it is also called silent thyroiditis or, in chronic cases, is also known as subacute lymphocytic thyroiditis [8]. Additionally, this thyroiditis is related to several inflammatory processes like insulitis and type 1 diabetes, as well as colitis, an autoimmune and inflammatory disease, where TLR3/4 overexpression may be present, supporting the possibility that environmental stressors, including systemic infections, might play a role [9]. In a review by Chong et al., 85 patients with high serum antithyroid antibody concentrations and encephalopathy were identified [10]. The study concluded that there is indeed evidence of a clinical syndrome constituted by the constellation of clinical symptoms, but cautioned that establishing a pathophysiologic link between a cerebral syndrome and antithyroid antibodies is not necessarily appropriate, as the pathogenesis is not well understood [10].

The mechanism underlying thyroid affection as a result of a viral infection is not fully understood, but several mechanisms have been proposed. Chiefly among them is molecular mimicry affecting self-antigens, which may induce polyclonal T-cell activation, consequently forming immune complexes and promoting the expression of major histocompatibility complex molecules in thyroid epithelial cells [11]. There have been reports of SARS-CoV-2 leading to thyroid dysfunction, usually weeks after symptom resolution, with a majority appearing to have thyroid autoimmunity at baseline. Most responded to a course of oral steroids, but persistence has also been reported [11,12]. Thyroid dysfunction is associated with higher viral loads and a more severe disease process with a worse prognosis. One retrospective study from Italy has shown a prevalence of 20.2% of thyroid disorders in hospitalized patients with severe COVID-19 infection [12].

The interaction between COVID-19 and thyroid disease can be multifaceted, especially in terms of the immune response [13]. Specifically, interleukin (IL)-6 plays a role in thyroid dysfunction by hampering the conversion of free T4 (fT4) to free T3 (fT3), accompanied by an inverse relationship with TSH levels, where higher levels correlate with poorer prognosis. Among cases developing thyroid dysfunction, thyrotoxicosis seemed to be more prevalent as compared to hypothyroidism (20% vs. 5%, respectively) and associated with a prolonged hospital course and higher mortality [13,14]. In most cases, TSH and fT3 levels dropped during the acute phase and remained low during convalescence. Overall, transient thyroid dysfunction could occur in COVID-19 patients and correlate with the degree of inflammation. Inflammatory reaction in COVID-19 infection is secondary to Th1/TH17 hypersensitivity, which may result in the onset of an autoimmune disorder including AIHA, immune thrombocytopenia (ITP), GBS, and Hashimoto's thyroiditis [14,15]. Severe COVID-19 infection is known to cause a cytokine storm through an increased Th1/Th17 immune response including excessive production of several proinflammatory cytokines, consisting of tumor necrosis factor-alpha (TNF-α) and IL-6 [12]. Therefore, patients with Hashimoto's thyroiditis and COVID-19 can be hypothesized to have an augmented immune response leading to an increased risk of cytokine storm and more severe COVID-19 infection. On the other hand, angiotensin-converting enzyme 2 (ACE2) has increased expression in thyroid cells than in lung cells, which is also a host-cell entry receptor for SARS-CoV-2 [16,17]. This can be attributed to a common pathogenic pathway. Thyroid glands of SARS-CoV-2 patients have shown severe pathologic injury in follicular epithelial cells with follicular distortion and collapse [18]. Autopsies of COVID-19 patients did not show viral particles in the thyroid gland. Therefore, an indirect immune mechanism for destructive changes and cellular apoptosis may be responsible for Hashimoto's thyroiditis [19]. COVID-19 has been associated with many diseases affecting the immune system including Hashimoto's thyroiditis, GBS, and Systemic lupus erythematosus (SLE)-like disease [20-21]. COVID-19 affects multiple organs including but not limited to the gastrointestinal tract, and cardiovascular and neurological systems [22-24]. Further research is necessary to evaluate for long-term effects of the novel coronavirus on thyroid function.

## Conclusions

We have described a case of Hashimoto's thyroiditis encephalopathy induced by the SARS-CoV-2 virus. There is existing evidence to suggest a link between thyroiditis and other viral entities, but there is a lack of data showing a link between COVID-19 and Hashimoto's thyroiditis. This report aims to inform about that possibility and add to a repertoire of clinical differentials when examining patients who present with encephalopathy and elevated serum antithyroid antibodies, in addition to the constellation of other possible clinical symptoms highlighted in this case presentation.
